# Regulatory Mechanisms of the NLRP3 Inflammasome, a Novel Immune-Inflammatory Marker in Cardiovascular Diseases

**DOI:** 10.3389/fimmu.2019.01592

**Published:** 2019-07-10

**Authors:** Na An, Yonghong Gao, Zeyu Si, Hanlai Zhang, Liqin Wang, Chao Tian, Mengchen Yuan, Xinyu Yang, Xinye Li, Hongcai Shang, Xingjiang Xiong, Yanwei Xing

**Affiliations:** ^1^Guang'anmen Hospital, China Academy of Chinese Medical Sciences, Beijing, China; ^2^Key Laboratory of Chinese Internal Medicine of Ministry of Education and Beijing, Dongzhimen Hospital Affiliated to Beijing University of Chinese Medicine, Beijing, China; ^3^Department of Acupuncture and Moxibustion, Shanxi University of Chinese Medicine, Taiyuan, China; ^4^Department of Acupuncture and Moxibustion, Beijing University of Chinese Medicine, Beijing, China

**Keywords:** NLRP3 inflammasome, cardiovascular diseases, immune-inflammatory, coronary atherosclerosis, myocardial ischemia/reperfusion

## Abstract

The nod-like receptor family pyrin domain containing 3 (NLRP3) is currently the most widely studied inflammasome and has become a hot topic of recent research. As a macromolecular complex, the NLRP3 inflammasome is activated to produce downstream factors, including caspase-1, IL-1β, and IL-18, which then promote local inflammatory responses and induce pyroptosis, leading to unfavorable effects. A growing number of studies have examined the relationship between the NLRP3 inflammasome and cardiovascular diseases (CVDs). However, some studies have shown that the NLRP3 inflammasome is not involved in the occurrence of certain diseases. Therefore, identifying the mechanism of action of the NLRP3 inflammasome and its potential involvement in the pathological process of disease progression is of utmost importance. This review discusses the mechanisms of NLRP3 inflammasome activation and the relationship between the inflammasome and CVDs, including coronary atherosclerosis, myocardial ischemia/reperfusion, cardiomyopathies, and arrhythmia, as well as CVD-related treatments.

## Introduction

**Figure 1:**
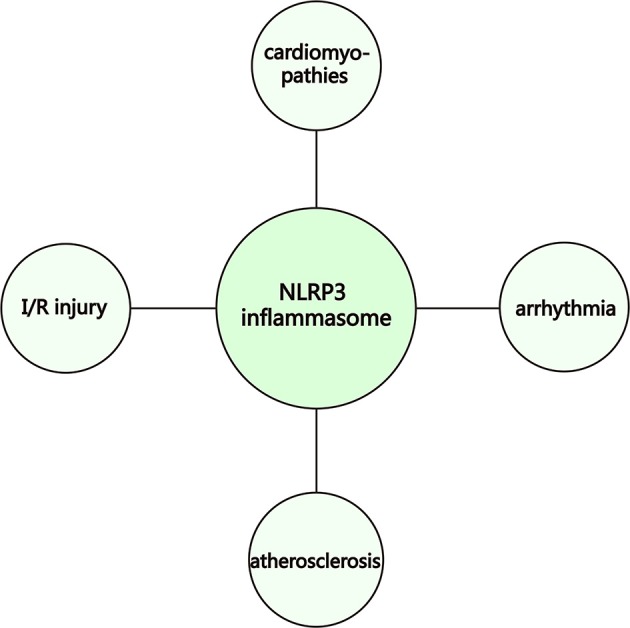
NLRP3 inflammasome and associated cardiovascular diseases.

Despite considerable improvements in preventing and treating cardiovascular diseases (CVDs), the lack of a comprehensive understanding of the mechanisms involved in the development of CVDs remains a principle factor implicated in global morbidity and mortality (1). For instance, in the United States of America, coronary heart disease (CHD), the most prevalent form of CVD, accounts for approximately half of the total number of CVD cases. It has been estimated that by the year 2035, more than 130 million adults will suffer from different forms of CVD, incurring a great cost as a direct result of increased medical expenses (2). Thus, gaining insight into the pathogenesis of CVDs and the search for new treatments are of utmost importance. Since the NLRP3 inflammasome can activated via a variety of different mechanisms and plays an important role in CVD development, the NLRP3 inflammasome is currently the most fully characterized inflammasom, and may be involved in disease progression (Figure 1). Inhibition of the NLRP3 inflammasome pathway may be a new approach for the prevention and/or treatment of CVDs. The NLRP3 inflammasome, which is composed of NLRP3, an adaptor protein apoptosis-associated speck-like protein (ASC), and caspase-1 (3), may be activated by intact pathogens, a large number of structurally diverse pathogen-associated molecular patterns (PAMPs), host-derived risk signals (danger-related molecular patterns, DAMPs), and environmental stimuli (4).

## NLRP3 Inflammasome and the Activation Mechanism

In order to detect PAMPs, the innate immune system scans the extracellular environment and endosomal compartments using pattern recognition receptors (PRRs), which include membrane-bound toll-like receptors (TLRs) and C-type lectins (CTLs) (4). Another group of receptors, the nucleotide-binding oligomerization domain-like receptors (NLRs), differ from TLRs and CTLs in that they are capable of recognizing both PAMPs and DAMPs (4). The NLR family has a central nucleotide binding and oligomerization (NACHT) domain that is typically surrounded by C-terminal leucine-rich repeats (LRRs) and N-terminal caspase recruitment (CARD) and pyrin (PYD) domains (5, 6). The NLR family is classified into three distinct subfamilies the NODs, the NLRPs (also known as NALPs), and the IPAF subfamily based on the NACHT domain (4). When NLRP3 is activated, ASC recruits pro-caspase-1 through the CARD domain to assemble an inflammasome. Subsequently, pro-caspase-1 is hydrolyzed into active caspase-1, a process which produces several proteins including pro-IL-1β and pro-IL-18 (7, 8). The protein pro-IL-1β is transcriptionally activated by the transcription factor nuclear factor kappa B (NF-κB). While pro-IL-18 is a fixed expression, which is expressed after cell activation, pro-inflammatory cytokine transcription, maturation and release (9, 10). The members of the IL-1β cytokine family that are activated by caspase-1 trigger the activation of other immune cells, such as neutrophils, at the site of infection or tissue damage, thereby promoting an inflammatory response (9).

Activation of the NLRP3 inflammasome occurs in two stages. Firstly, signaling through the TLR and NF-κB pathways initiate the transcriptional synthesis of various inflammatory factor precursors such as pro-IL-1β (11, 12). Secondly another signal is transduced by multiple PAMPs and DAMPs forming the NLRP3 inflammasome macromolecular complex which is assembled by NLRP3, ASC, and pro-caspase-1 (13, 14).

The NLRP3 inflammasome can be activated by multiple mechanisms, including reactive oxygen species (ROS), which are essential inflammation signals. In this mode, changes in the concentration of ROS are detected by thioredoxin interacting protein (TXNIP) which then separates from thioredoxin (TRX) and binds to NLRP3 effectively activating it (15, 16). Activation may also occur through adenosine triphosphate (ATP) via the P2X7 receptor, a characteristic non-selective ATP-gated cationic channel. Extracellular signaling molecules, including ATP, other nucleotides and nucleosides, operate through a complex purinergic signaling network that consists of the P2X7 and other membrane receptors, as well as ectoenzymes (17). When ATP is released into the extracellular medium, elevated levels of ATP are detected by the P2X7 receptor, which in turn induces the production of ROS and the activation of NF-κB (18). In an acute myocardial infarction (AMI) trial, P2X7 was revealed to be effective at activating NLRP3, triggering an inflammatory response which negatively impacts prognosis (19, 20). At the same time, activation of the P2X7 receptor allows for small positive ions to pass through the cell membrane resulting in potassium outflow. The NLRP3 inflammasome can also be activated by a decrease in intracellular potassium concentration (21). Rupture of lysosomes is also a signal that leads to the activation of NLRP3. Certain PAMPs and DAMPs are sensed by lysosomes, causing lysosome damage and loss of integrity through phagocytosis. The hydrolyzed lysosomes are released into the cytoplasm, and the NLRP3 inflammasome is activated by indirectly sensing lysosomal damage (22, 23). Many different types of post-translational modifications are an indispensable part of NLRP3 inflammasome assembly, including ubiquitylation, phosphorylation, deubiquitylation, and dephosphorylation (24). More specifically, linear ubiquitination is required for assembly of the NLRP3/ASC inflammasome (25). NLRP3 inflammasome formation also requires phosphorylation of ASC by the spleen tyrosine kinase (Syk) (26). BRCA1/BRCA2-containing complex subunit 3 (BRCC3), a deubiquitinating enzyme, is critical for NLRP3 activity by promoting its deubiquitination (27). Phosphatase 2A (PP2A) also promotes NLRP3 activation via dephosphorylating NLRP3 PYD (28).

In addition, the NLRP3 inflammasome is also associated with autophagy and mitochondrial autophagy (mitophagy). Knockout of CD38 and lysosomal V-ATPase-inhibition induced lysosomal dysfunction and autophagy disorder can result in activation of NLRP3 inflammasome (29). The NLRP3 inflammasome can also be activated, in a way that inhibits autophagy, by knocking out sirtuin (sirt) 3 (30). Furthermore, overexpression of sirt3 reduced the activation of NLRP3 inflammasome in THP-1 cells, mainly due to a decrease in mitochondrial ROS production induced by palmitic acid and recovery of autophagy (30). CVL, an autophagy inducer, also inhibits NLRP3 inflammasome activation via the sirt1/autophagy signaling pathway (31). In contrast, the NLRP3 inflammasome can also inhibit autophagy through caspase-1-mediated TRIF-cleavage (32). Mitophagy is a process of degradation and recycling through autophagy after mitochondrial damage that acts as a negative regulator of NLRP3 inflammasome activation (33). When mitophagy is triggered via the AMPK/ULK1 signaling and PIK3CA-AKT1-MTOR-RPS6KB1 pathways, the NLRP3 inflammasome is inhibited (34, 35). NOD2-RIPK2 is a related signaling pathway, which is also involved in autophagy, mitophagy and the production of IL-18, and specifically regulates mitophagy (36).

## NLRP3 and CVDs

**Figure 2:**
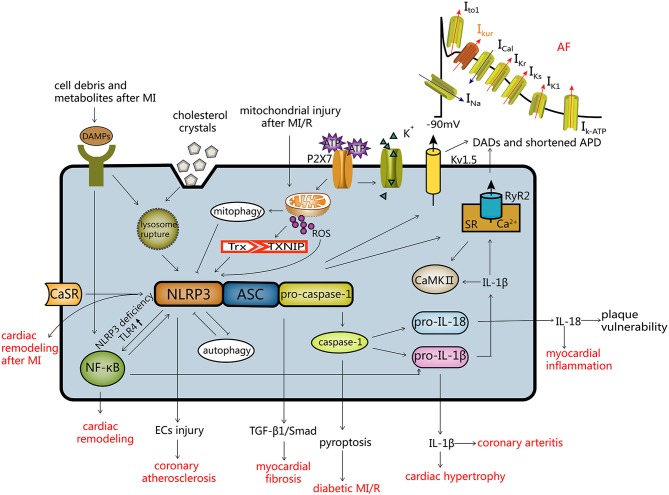
Activation modes of the NLRP3 inflammasome and the regulatory mechanism of CVDs. Activation of NLRP3 (1) ROS mode; (2) the ATP mode; (3) lysosome mode. Autophagy and the NLRP3 inflammasome inhibit each other, and mitochondrial autophagy inhibits the NLRP3 inflammasome. The NLRP3 inflammasome and CVDs: (a) after activation of the NLRP3 inflammasome, endothelial cells are damaged resulting in coronary atherosclerosis; (b) myocardial fibrosis is induced by the TGF-β1/Smad pathway; (c) caspase-1 mediated pyroptosis, leading to diabetic MI/R; (d) IL-18 and IL-1β can cause myocardial inflammation, coronary arteritis and myocardial hypertrophy; (e) the NLRP3 inflammasome leads to atrial fibrillation by influencing Kv1.5 and SR Ca^2+^ leakage; (f) NLRP3 deficiency induces cardiac remodeling via the TLR4/NF-κB pathway; (g) activation of the CaSR/NLRP3 inflammasome pathway leads to cardiac remodeling after MI. DADs, delayed after depolarization; APD, action potential duration.

Activation of the NLRP3 inflammasome has been shown to contribute to regulation of pathogenesis of a number of CVDs (Figure 2).

### NLRP3 and Coronary Atherosclerosis

Atherosclerosis often results in thrombosis or arterial stenosis which may lead to a variety of ischemic diseases including myocardial infarction (MI), ischemic stroke, further tissue ischemia, and in severe cases, can affect the quality of life (37). Atherosclerotic plaque formation often involves endothelial dysfunction and other complex processes, and oxidative stress is believed to increase the prevalence of atherosclerosis (38). The NLRP3 inflammasome has also been linked to the onset of coronary atherosclerosis. The NLRP3 inflammasome in subcutaneous adipose tissue (SAT) from patients who underwent heart device implantation and coronary angiography, may be involved in the development of atherosclerosis and is directly connected with the severity of coronary atherosclerosis (39).

The relationship between the NLRP3 inflammasome and coronary atherosclerotic heart disease through cholesterol crystals/monosodium glutamate (MSG), downstream factors and endothelial cell (EC) injury is summarized below.

#### Cholesterol Crystals/MSG

Cholesterol crystals are a danger signal that can drive sterile inflammation, such as atherosclerosis, by interacting with neutrophils (40). They appear at the earliest stages of diet-induced atherosclerosis, almost simultaneously with subcutaneous immune cells (41). During disease progression, macrophages can phagocytize and accumulate cholesterol crystals through the CD36 receptor and activate the inflammasome through lysosomal damage (41, 42). NLRP3 in the cytoplasmic matrix of macrophages and foam cells is involved in the cleavage of cholesterol crystals (43). Early atherosclerosis and IL-18 levels notably decreased after using a high-cholesterol diet when the NLRP3 inflammasome components and its important downstream factors, including NLRP3, ASC, and IL-1α/β, were simultaneously knocked out in ldlr-deficiency mice (41). NF-E2-related-2 (Nrf2), a transcription factor, plays an important role in endogenous antioxidant stress systems (44). Nrf2 inhibits activation of the NLRP3 inflammasome through the Trx1/TXNIP complex (45). Cholesterol crystals induce Nrf2-related signaling pathways, including the caspase-1-independent IL-1 signaling and NLRP3/caspase-1-dependent IL-1 pathways, leading to atherosclerosis. Diet-induced atherosclerosis was lower in Nrf2^−/−^Apoe^−/−^ than in heterozygous Nrf2^+/−^Apoe^−/−^ mice, but neutralization of IL-1α and IL-1β did not reduce atherosclerosis in Nrf2^−/−^Apoe^−/−^ (46). One of the activation mechanisms of the NLRP3 inflammasome is lysosomal phagocytosis of exogenous activators, including crystals and particulate structures, when the substances released after lysosomal injury are sensed by the NLRP3 inflammasome (4). Similarly to cholesterol crystals, MSG crystals can act as DAMPs to activate the NLRP3 inflammasome and cause inflammatory responses, which eventually lead to gout rather than hyperuricemia, increasing the risk of CHD (47).

#### IL-18, IL-1β, and Caspase-1

IL-18 is a pro-inflammatory responsive factor which plays a vital role in the inflammatory response (48). IL-18 is known to be an interferon (IFN)-γ-inducible factor with strong activity on macrophages and T cells, which play a crucial role in the occurrence and development of atherosclerosis (49, 50). IL-18 is independent of other risk factors and inflammatory markers, which are associated with the occurrence of CHD in the Prospective Epidemiological Study of Myocardial Infarction (51). The IL-18 levels are significantly elevated in plaques and IL-18 mRNA transcription is associated with the development and vulnerability of atherosclerotic plaques. IL-1β acts primarily extracellularly as a soluble mediator, which acts on a variety of cells and organs, including vascular endothelial, smooth muscle cells, and macrophages (52). IL-1β acts on the outside cells to increase the levels of leukocyte adhesion molecules and thrombosis-inducing substances. In the case of IL-1α or IL-1β inducing themselves or each other, a positive feedback loop pertains. IL-1 receptor I (IL-1RI) transduces IL-1β signaling. The formation of the IL-1 receptor complex heterodimer results in the generation of the IL-1β precursor. And then the assembled NLRP3 inflammasome results in the formation of the active caspase-1. Active caspase-1 processes the IL-1β precursor in the cytosol or in the secretory lysosome, results in the generation and secretion of IL-1β (10, 52). The type II IL-1 receptor (IL-1RII) binds IL-1 but does not transduce a signal, and acts as a sink for IL-1β and has been termed a “decoy” receptor because of its anti-inflammatory property. A hyperlipidemic condition may exacerbate the indirect effect of saturated fatty acids due to a decrease of IL-1RII in monocytes, which may augment the expression and release of adhesion molecules in vascular endothelial cells, result in triggering of the onset of CVDs (53). The level of NLRP3 in the CHD group was distinctly higher than that in the non-CHD group, and the levels of IL-1β and IL-18 were positively correlated with the level of the NLRP3 inflammasome expression, producing the chronic inflammation involved in the pathogenesis of CHD (54). Caspase-1 is an enzyme that induces IL-18 and IL-1β maturation, and knockdown of caspase-1 can reduce atherosclerotic lesions in ApoE^−/−^ mice (55) and significantly reduce the plaque area (56). ATP-binding cassette transporters A1 and G1 (Abca1/g1) deficiency leads to cholesterol deposition, which actives NLRP3 inflammasome. IL-18 and IL-1β levels were elevated in the group with an Abca1/g1 deletion. This was unlike the NLRP3 or caspase-1 deletion groups, suggesting that in the absence of Abca1/g1, the NLRP3 inflammasome was activated and caspase-1 cleavage was increased (56).

These results indicated that factors downstream of the NLRP3 inflammasome are involved in the pathogenesis of coronary atherosclerotic heart disease.

#### Vascular Endothelial Cell Injury in Coronary Heart Disease

The EC injury and inflammatory responses also play a role in the formation of coronary atherosclerosis (57, 58). EC promote apoptosis, reduces proliferation rate, and increases the density of inflammatory proteins after senescent injury. This damage is particularly prevalent in elderly patients presenting with CVDs (59, 60). Under the stress of CHD, microRNA-22 (miR-22) can reduce the apoptosis rate, increase cell activity and reduce EC damage. MiR-22 was found to reduce the level of pro-inflammatory cytokines by inhibiting the NLRP3 inflammasome pathway, thereby protecting EC in rats afflicted with CHD (61). This indicates that the NLRP3 pathway can damage EC and cause CHD.

#### Atherosclerosis Progresses Independently of the NLRP3 Inflammasome

Surprisingly, it was revealed that there were no differences in the progression of atherosclerosis and plaque stability in NLRP3/ApoE, ASC/ApoE, and caspase-1/ApoE double knockout mouse models (Table 1) (62). These results suggest that atherosclerosis in ApoE^−/−^ mice model progresses independently of the NLRP3 inflammasome. There may be two reasons for this discrepancy. Firstly, IL-1α played an important role in atherosclerosis development in the ApoE^−/−^ mice model (62). One study showed that the absence IL-1α significantly decreased atherosclerotic plaque area in a high-fat diet C57BL/6 mouse model, and the absence of IL-1β did not reduce atherosclerosis development in a statistically significant manner (63). In another study, IL-1α deficiency reduced the aortic sinus lesion area compared with IL-1β in ApoE^−/−^ mice (64). As active IL-1α can be produced in the absence of the NLRP3 inflammasome through calpain-mediated processing (65), deficiency of the NLRP3 inflammasome would not impair the development of atherosclerosis (62). Secondly, in the same high-cholesterol (1.25%) diet, ApoE^−/−^ mice had higher plasma cholesterol and more serious pathologic changes (66), thus minor contributions of genes that potentially result in atherosclerosis might be missed in the prior model (62).

**Table 1:** Mechanisms of KO/KI animal models for NLRP3 inflammasome pathway related genes.

**Genotype**	**Animal**	**KO or KI**	**Cardiovascular diseases**	**Mechanism of action**	**References**
NLRP3	Mice	KO	Atherosclerosis	Reduced plasma levels of IL-18 and decreased atherosclerotic lesion size	(41, 56)
	Mice	KO	Atherosclerosis	Not critically implicated in atherosclerosis progression	(62)
	Mice	KO	Cardiac remodeling	Increased TLR4 expression and deteriorated cardiac remodeling via regulation of the TLR4/NF-κB pathway	(89)
	Mice	KI	AF	Developed spontaneous premature atrial contractions and inducible AF; exhibited ectopic activity, abnormal sarcoplasmic-reticulum Ca^2+^-release, AERP shortening and atrial hypertrophy	(103)
ASC	Mice	KO	Atherosclerosis	Decreased early atherosclerosis and inflammasome-dependent IL-18 levels	(41)
	Mice	KO	Atherosclerosis	Did not influence atherosclerosis progression	(62)
Caspase-1; Caspase-1/11	Mice	KO	Atherosclerosis	Decreased the number of lesion-associated cells expressing major histocompatibility complex class II and reduced lesion-associated IFN-γ expression Reduced neutrophil accumulation and neutrophil extracellular trap formation in atherosclerotic plaques and decreased plasma IL-18 levels	(55, 56)
	Mice	KO	Atherosclerosis	No effect on the development of atherosclerosis	(62)
IL-1α/β	Mice	KO	Atherosclerosis	Decreased plasma levels of IL-18 as well as lesional area	(41)
IL-18	Mice	KO	Cardiac hypertrophy	Blunted the pro-hypertrophic signaling pathway	(91)
Nrf2	Mice	KO	Atherosclerosis	Highly protected against diet-induced atherogenesis by cholesterol crystal-induced IL-1 responses	(46)
CD38	Mice	KO	Atherosclerosis	Led to lysosome dysfunction and autophagy derangement, and increased colocalization of NLRP3 vs. ASC or caspase-1 enhanced IL-1β accumulation and caspase-1 activity	(29)

*NLRP3, Nod-like receptor family pyrin domain containing 3; TLR, Toll-like receptor; ASC, adaptor protein apoptosis-associated speck-like protein; IL, interleukin; IFN, interferon; Nrf2, NF-E2-related-2; AF, Atrial fibrillation; KO, knockout; KI, knock-in*.

### NLRP3 and Myocardial Infarction/Reperfusion

After the onset of AMI, ischemic injury, cell death and the associated cell debris and metabolites act as DAMPs thereby activating the inflammasome leading to an inflammatory response (67, 68). In the clinical treatment of AMI, reperfusion can reduce ischemic injury and infarct size. However, after reperfusion, ROS levels increase, and pro-apoptotic proteins are released as a result of mitochondrial injury (69). Therefore, reperfusion injury can also affect infarct size. Using novel small molecule inhibitors of the NLRP3 inflammasome can reduce myocardial infarct area and maintain cardiac function in animal models of MI (70). The mechanism of NLRP3 in ischemia/reperfusion (I/R) may be related to TXNIP, pyroptosis, and EC injury. But NLRP3 may not play a role in mouse models lacking surgical trauma. The closed-chest mouse model of myocardial infarction/reperfusion (MI/R) reduces the inflammatory response induced through the surgical stress of the frequently-used open-chest model. NLRP3 protein was detected in the I/R-hearts of the open-chest model, but not in the I/R-hearts of the closed-chest model indicating that NLRP3 expression in the I/R-hearts of the open-chest model is mainly due to acute surgical stressors and not related to acute cardiac I/R (71).

#### TXNIP

In a new mechanism of MI/R injury, TXNIP activates the NLRP3 inflammasome in cardiac microvascular ECs via ROS in *in vitro* experiments (72). As the concentration of intracellular ROS increases, this complex dissociates and TXNIP binds to the LRR region of NLRP3, which results in the activation of the NLRP3 inflammasome (73). Intramyocardial TXNIP siRNA injection attenuated infarct size and NLRP3 activation in MI/R mice showing that the interaction between TXNIP and NLRP3 was enhanced in ischemic hearts (72). A similar mechanism exists in cerebral I/R injury where Nrf2 negatively regulates activation of the NLRP3 inflammasome. Nrf2 activates the Trx1 gene, prompting the formation of a compound consisting of Trx1 and TXNIP, thereby inhibiting TXNIP activation of the NLRP3 inflammasome. These results indicate that TXNIP activation of the NLRP3 inflammasome may be one of the activation mechanisms in the case of MI/R injury (45).

#### Pyroptosis

The NLRP3 inflammasome, in addition to IL-18 and IL-1β, not only plays pivotal roles in inflammation in cardiac fibroblasts and circulating inflammatory cells, but also in the occurrence of pyroptosis, caspase-1 dependent programmed cell death (74–76). Pyroptosis is a newly discovered form of cell death, which is driven by non-infectious factors, including host factors produced during MI (76, 77). The primary ischemic injury and the subsequent mitochondrial damage result in activation of the NLRP3 inflammasome in the heart, inducing inflammatory cell pyroptosis in cardiomyocytes, further increasing myocardial injury and infarct size (68, 78). In diabetic rats, pyroptosis mediated by the NLRP3 inflammasome, which was activated by ROS, played an important role in MI/R injury. Inhibition of NLRP3 could attenuate MI/R injury, and in the presence of MI/R, caspase-1 and IL-1β were positively correlated with infarct size and the degree of injury. These results show that NLRP3-induced pro-inflammatory programmed cell death is the initial response to MI/R injury in diabetes (79).

#### Vascular Endothelial Cell Injury in MI

In the build up to MI/R, EC damage is caused by an anoxic environment and ROS plays an important role in vascular endothelial injury during cardiovascular pathology (80, 81). EC injury and the inflammatory response were improved through regulation of the NLRP3 pathway, which is related to miR-495. MiR-495 can inhibit cell apoptosis and reduce the mRNA and protein levels of NLRP3 as well as downstream factors in mice with MI/R injury (82). In another study, miR-495 was thus shown to not only contribute to atherosclerotic lesion formation, but also plaque stability in ApoE^−/−^ mice. Inhibition of miR-495 reduced intimal hyperplasia and decreased accelerated atherosclerosis and plasma cholesterol levels (83). A recent study revealed that activation of the NLRP3 inflammasome occurred after cerebral I/R, suggesting that initially microglia are the main source of activated NLRP3 inflammasomes, and in the later stages, the NLRP3 inflammasome is activated within neurons and vascular EC (84). This raises new questions on the relationship between MI/R and the NLRP3 inflammasome.

### NLRP3 and Cardiomyopathies

In addition to the aforementioned diseases, the NLRP3 inflammasome may also be involved in the pathological mechanism of cardiomyopathies, including cardiac remodeling and cardiac hypertrophy. However, excessive inhibition of the NLRP3 inflammasome may also have adverse effects on the disease.

#### Cardiac Remodeling

Cardiac remodeling generally features changes in cardiac structure, shape, and function. These alterations are an adaptive response to maintain cardiac function; nevertheless, in the context of sustained stress and over a period of time, these changes become maladaptive, and the heart eventually fails (85, 86). The calcium sensing receptor (CaSR)/NLRP3 inflammasome through the phospholipase C-inositol phosphate 3 (PLC-IP3) pathway in M1 macrophages plays a pivotal role in accelerating cardiac remodeling after MI in rats, including promoting cardiac fibroblast phenotypic transversion and increasing collagen as well as extracellular matrix secretion (87). In mice where Ca^2+^/calmodulin-dependent protein kinase II δ (CaMKIIδ) was selectively deleted from cardiomyocytes and in floxed control mice subjected to transverse aortic constriction (TAC), inflammation and NLRP3 inflammasome activation initiated in response to pressure overload by CaMKIIδ signaling in cardiomyocytes are essential for adverse cardiac remodeling (88). In addition, NLRP3 deficiency accelerates cardiac hypertrophy, fibrosis, as well as inflammation responses induced by pressure overload in a cardiac remodeling mouse model. NLRP3 deficiency also increased the level of TLR4 expression, which results in adverse cardiac remodeling (89).

#### Cardiac Hypertrophy

In prospective studies, IL-18 is a strong independent predictor of coronary artery disease (51, 90). IL-18 plays a key role in the hypertrophic response, with down-regulation of hypertrophy-related genes seen in IL-18 knockout mice (91). In a rabbit model, IL-18 expression also increased significantly during pressure loading, with sustained upregulation of IL-18 in response to transaortic constriction (92). The levels of the NLRP3 inflammasome were notably increased in mice with TAC and were involved in the increase in inflammatory mediators and profibrotic factor production, leading to myocardial fibrosis, cardiomyocyte hypertrophy, and impaired cardiac function (93). When the oxidative stress system of myocardial cells is activated, accumulated ROS and Ca^2+^ are released to the cytoplasm, resulting in changes in mitochondrial membrane potential, leading to apoptosis of myocardial cells (94). Pirfenidone improved TAC-induced left ventricular hypertrophy and myocardial fibrosis in a mouse model through inhibiting NLRP3 inflammasome assembly, and regulating the NLRP3-IL-1β signaling pathway in both the ROS-dependent and ROS-independent pathways (95). Moreover, NF-κB and NLRP3 were related to receptor for advanced glycation end products (RAGE)-induced cardiomyocyte hypertrophy that may be induced via activation of the NF-κB-NLRP3-IL-1β signal pathway in H9C2 cells (96). These studies revealed that the signaling pathways related to the NLRP3 inflammasome may be involved in the pathological process of myocardial hypertrophy.

### NLRP3 and Arrhythmia

The mechanism of action of the NLRP3 inflammasome in the pathogenesis of arrhythmia after heart failure and atrial fibrillation (AF) is summarized below.

#### Arrhythmia After Heart Failure

Patients with heart failure often experience changes in electrical remodeling, which result in arrhythmia (97). TLR2 and NLRP3 inflammasome activation in heart macrophages induce the production of IL-1β in diabetes mellitus mice (98). IL-1β then decreased the density of L-type Ca^2+^ (I_CaL_), implicating ROS signaling and protein kinase C activation and resulting in the development of arrhythmia and heart failure (99). During heart failure, persistent inflammatory stimulation leads to collagen accumulation and myocardial fibrosis that aggravates the condition (100). The myocardial fibroblasts then in turn induce inflammatory mediator production forming a vicious cycle (101). The expression of NLRP3 and ASC as well as inflammasome assembly were inhibited, and the NLRP3-transforming growth factor β1 (TGFβ1)-Smad pathway was blocked, in mice when mouse cardiac fibroblasts were treated with anti-fibrotic drugs (102).

#### Atrial Fibrillation

The activity of NLRP3-inflammasomes is markedly enhanced in the atrial cardiomyocytes of AF patients and dogs with atrial tachycardia pacing. A cardiomyocyte-specific knock-in mouse model expressing constitutively active NLRP3, exhibited spontaneous premature atrial contractions and inducible AF (103). Overactive NLRP3-signaling in cardiomyocytes enhances the expression of ryanodine receptor type-2 (RyR2), leading to increased protein expression and abnormal release of sarcoplasmic reticulum (SR) Ca^2+^. Meanwhile, the transcription of Kcna5 was enhanced, which resulted in augmented Kv1.5-current (I_kur_) that abbreviates the atrial effective refractory period, creating a reentry substrate (103).

## New Therapies Related to the NLRP3 Inflammasome

The small molecule inhibitor, MCC950, is selective for the NLRP3 inflammasome and does not inhibit other inflammasomes, such as NLRP1 and NLRP4, nor does it affect other immune responses (104). MCC950 has the potential to prevent diseases associated with the NLRP3 inflammasome, including small vessel disease, stroke in diabetic patients (105), hypertension, kidney damage (106), as well as CVDs (107). Another NLRP3 inflammasome inhibitor, CY-09, is binds the NACHT domain which limits NLRP3 oligomerization and assembly of the inflammasome. Additionally, CY-09 inhibits NLRP3 activation of ATPase, but does not affect NLRP1 ATPase activation, suggesting that CY-09 is specific in its inhibition of the NLRP3 inflammasome (108). CY-09 significantly reduces platelet aggregation by affecting the threshold concentration of collagen, as well as impaired clot contraction in platelets (109).

In clinical trials of a canakinumab anti-inflammatory thrombosis outcomes study (CANTOS), canakinumab inhibited the expression of IL-1 related genes. Canakinumab may therefore reduce the incidence of CVDs and may, in the future, become a new treatment for the secondary prevention of CVDs and other diseases (110). Subsequent clinical trials revealed that the patients receiving canakinumab exhibited a decrease in high-sensitivity C-reactive protein compared with the placebo group. Changes in low-density lipoprotein cholesterol, high-density lipoprotein cholesterol and triglycerides were not significant, suggesting that canakinumab targets the IL-1β protein, reduced CVD and was not associated with lowering blood lipid levels. The most effective dose of canakinumab was 150 mg every 3 months (111). In coronary artery disease, statins especially atorvastatin, can reduce expression of the NLRP3 inflammasome as well as downstream factors IL-1β and IL-18. In contrast, the effect of rosuvastatin is not obvious. Statins have inhibitory effect on endothelial dysfunction which accounts for their mechanism of action (54). Statins may also treat I/R injury by inhibiting the NLRP3 inflammasome, although further research is required to explore this concept (112). Colchicine displayed a greater effect on CVDs. After MI in mice, colchicine reduced acute inflammation in infarct areas, improved survival, prevented heart failure, reduced ventricular remodeling and maintained stability in cardiac function (113). Colchicine was capable of reducing myocardial injury induced by elevated levels of IL-1β in the infarcted area from early reperfusion (114). Colchicine also inhibits the NLRP3 inflammasome and caspase-1 and the associated inflammatory response and matrix metallopeptidase (MMP) expression (including MMP2 and MMP9) (113). Colchicine significantly reduces the prevalence of MI in patients with gout (115). For patients with stable angina pectoris, taking colchicine significantly reduces the incidence of acute coronary syndrome and other symptoms (116). The addition of low doses of colchicine (0.5 mg/d) to standard drugs such as statins produced better results, significantly reducing the incidence of cardiovascular events (116). In addition, pigment epithelium-derived factor (PEDF) is accepted as another method for AMI treatment. In a rabbit model, PEDF reduced the activation of the NLRP3 inflammasome, supposedly by inhibiting mitochondrial division through the pigment epithelial-derived factor receptor/calcium-independent phospholipase A2 (PEDFR/iPLA2), indicating that PEDF can also be used as a treatment strategy for ischemic diseases, including AMI and MI/R injury (117). Since total flavones (TA) can inhibit cell death and reduce oxidative stress and inflammation, they have a definite role in the treatment of CVDs. Studies in I/R mice have revealed that TA inhibits the activation of the NLRP3 inflammasome and has a positive therapeutic effect on I/R (118). Triptolide (TP) alleviates cardiac hypertrophy induced by isoproterenol (ISO) in mice (119). In addition, low-dose TP improves mouse cardiac fibrosis induced by ISO (*in vivo*) or angiotensin II (*in vitro*) through blocking NLRP3 inflammasome assembly, and inhibits the activation of the NLRP3-TGFβ1-Smad pathway (102).

These studies revealed that the use of NLRP3 inflammasome inhibitors and blocking the relevant pathways represents new treatment strategies for NLRP3 inflammasome related diseases. However, the effectiveness of these new treatment methods has to be validated, not only through animal studies, but also through clinical trials (Table 2).

**Table 2:** New therapies related to the NLRP3 inflammasome.

**Inhibitors of the NLRP3 inflammasome**	**Mechanism of action**	**References**
MCC950	Inhibited the NLRP3 inflammasome selectively	(104–107)
CY-09	Binds to the NACHT domain which limits NLRP3 oligomerization and assembly of the inflammasome; inhibited NLRP3-mediated activation of ATPase selectively	(108, 109)
Canakinumab	Inhibited the expression of IL-1 associated genes; targeted for IL-1β anti-inflammatory therapy	(110, 111)
Statins	Reduced the expression of the NLRP3 inflammasome as well as the downstream factors IL-1β and IL-18	(54, 112)
Colchicine	Reduced acute inflammation in infarct areas, improved survival, inhibited heart failure, reduced ventricular remodeling and maintained stability in cardiac function	(113–116)
Colchicine and statins	Significantly reduced the incidence of cardiovascular events	(116)
PEDF	Inhibited mitochondrial division through PEDFR/iPLA2	(117)
TA	Inhibited cell death and reduced oxidative stress and inflammation	(118)
TP	Inhibited the expression of NLRP3 and ASC as well as inflammasome assembly, and blocked the NLRP3-TGFβ1-Smad pathway	(102, 119)

*PEDF, pigment epithelium-derived factor; TA, total flavones; TP, triptolide*.

## Concluding Remarks

The discovery of the NLRP3 inflammasome has enriched our understanding of the pathogenesis of CVDs and other diseases. However, some experimental results demonstrate that the NLRP3 inflammasome has no influence on the occurrence of disease, so the mechanism of action by which these various diseases occur requires further attention. Moreover, research on the inhibition of NLRP3 inflammasome activity is equally important and provides novel targets for the treatment of CVDs. The current review has demonstrated that canakinumab, MCC950, colchicine, statins, TA, TP, and other downstream factor inhibitors treat disease by inhibiting the NLRP3 inflammasome pathway. It is worth noting that the inhibition of the NLRP3 inflammasome may also have adverse effects, and therefore, the effect of inhibition of the NLRP3 inflammasome needs to be continuously explored.

## Author Contributions

YX and YG designed the manuscript. NA and XY drafted the manuscript. XX and HS revised the manuscript. HZ, ZS, LW, and MY searched the literature. CT and XL aided in the design of the illustrations. All authors approved the manuscript for publication.

### Conflict of Interest Statement

The authors declare that the research was conducted in the absence of any commercial or financial relationships that could be construed as a potential conflict of interest.
